# Therapeutic comparison in psychological capital

**DOI:** 10.3389/fpsyt.2023.1114170

**Published:** 2023-08-07

**Authors:** Gloria Hongyee Chan

**Affiliations:** Department of Social and Behavioral Sciences, City University of Hong Kong, Kowloon Tong, Hong Kong SAR, China

**Keywords:** hikikomori, narrative therapy, cognitive-behavioral therapy, play therapy, intervention, outcomes

## Abstract

**Aim:**

This study aimed to investigate and compare the therapeutic outcomes of psychological capital between narrative therapy, cognitive-behavioral therapy, and play therapy in the context of hikikomori.

**Methods:**

This study included 502 hikikomori. Correlation analysis was performed to investigate the relationship between the three forms of therapy and psychological capital, while one-way ANOVA and independent samples *t*-tests were performed to determine the differences in the outcomes of psychological capital between the three forms of therapies.

**Results:**

Results indicated that all three forms of therapy were significantly positively related to psychological capital. Moreover, while cognitive-behavioral therapy performed better in psychological capital (overall score) than the other two, cognitive-behavioral therapy performed better in the subscales “self-efficacy” and “resilience,” while narrative therapy performed better in the “hope” and “optimism” subscales. Also, combining features of play therapy helped enhance the outcomes of narrative therapy and cognitive-behavioral therapy on psychological capital.

**Conclusion:**

Owing to the varied outcomes of psychological capital among different therapies, the differential use of therapies to deal with the unique needs resultant of hikikomori helps achieve optimal results.

## 1. Introduction

Hikikomori, who are typically young people, are referred to as those who have retreated from social connections and social participation for at least 6 months (1, 2). Reviewed by existing literature, this group of youth experience various negative life events such as parental issues, bullying, difficulty in fitting into society, and stigma related to prolonged social withdrawal [e.g., (3–6)]. Not being able to deal with these negative experiences, they develop “a strong sense of failure and inadequacy,” disempowerment, a low level of self-confidence, and a sense of hopelessness for the future [(3, 7), p. 1207; (6)]. This contributes to their engagement in social withdrawal behavior as a coping strategy (3, 6). This suggests that merely emphasizing the removal of the youth’s hidden behavior through the enhancement of social skills and relationships, their re-engagement into society, and resumption of employment to help them become functioning individuals (e.g., (7–9)) is inadequate. Instead, nurturing their psychological capital (PsyCap), a positive psychological state characterized by self-efficacy, hope, optimism, and resilience (10), is crucial. Psychological capital (PsyCap) is characterized by one’s “resources” and “capacities” which make them “positively appreciate daily life events and enlarge their likelihood of success by entrusting persistence and striving” [(11), pp. 678–679]. Having equipped with these inner resources, hikikomori will be more able to cope with the challenges and adversities in life adaptively, and have a positive sense of well-being. This may help enhance the readiness of hikikomori to re-engage into society.

Owing to the importance of psychological capital for hikikomori, this study sought to investigate the therapeutic outcomes of psychological capital among three kinds of therapy: cognitive-behavioral, narrative, and play therapies, which were implemented on hikikomori based on their unique needs and situations underlying their social withdrawal state [e.g., (3, 4, 7)]. Cognitive-behavioral therapy helps deal with hikikomori’s maladaptive thoughts and fosters their adaptive behavioral coping capabilities (12, 13); narrative therapy helps enhance hikikomori’s sense of self (14); play therapy helps enhance hikikomori’s social skills, sense of teamwork, cognitive capabilities, and leadership qualities that equip them with the requisite capabilities to handle issues in their daily lives (15). Such therapeutic goals correspond well with the enhancement of psychological capital in terms of: (1) setting personal goals and developing ways to achieve them; (2) experiencing mastery of tasks; (3) developing capabilities for positive appraisal of events; and (4) developing strategies to cope with setbacks (16, 17). In this study, the relationship between psychological capital and these three types of therapies will be investigated, followed by a simultaneous comparison of these therapies in the therapeutic outcomes of psychological capital. This is expected to generate corresponding practice implications.

## 2. Literature review

### 2.1. The life circumstances of hikikomori

Reviewing the existing literature, hikikomori suffers from a range of negative experiences in life, such as family dynamics, academic competition, career competition, and the severe stigma surrounding hikikomori [e.g., (3, 18, 19)]. Regarding family issues, it is described that hikikomori have the typical experiences of having mothers who impose great academic expectations on them to attain the desired goals of entering prestigious schools and pursing distinguished career paths (3, 7), or emotionally neglectful parents, which results in the youth adapting by suppressing their “original identities and authentic feelings” [(4), p. 182]. Regarding school experiences, inability to fit into the education system, inability to adapt to the peer group, and bullying are events related to social withdrawal (20, 21). Regarding career experiences, “negative appraisals and thinking” toward the career life and future and seeing themselves as “unable to secure a job” is a negative experience for hikikomori (6, 22). Moreover, these negative experiences can occur simultaneously, resulting in the youth suffering from severe negative labeling from society as well as significant others (e.g., parents and teachers) such as “lazy” and “useless” [(3), p. 132]; such censuring over their deviance from being normal and the inability to fit into the societal standards in terms of academic and career achievements causes a severe marginalization of these youth, increasing their difficulty in securing their position in mainstream society (3). Having internalized these negative experiences, these youth develop negative identities of “losers” and “failures” and use prolonged social withdrawal as a coping strategy [(3), p. 132]. In other words, engaging in prolonged social withdrawal and becoming hikikomori is a way to adapt to the life challenges and alleviate stress (23). Regarding support and intervention for hikikomori, instead of merely focusing on their behavioral aspect through helping these youth build social skills, social relationships, and re-enter the labor market [e.g., (7–9)], it is more important to be concerned about the psychological side of hikikomori, such as the inner self, perception of life events, and coping resources. As long as they have restored their self-confidence and are equipped with resources and adaptive methods to cope with difficulties in life, the impact of negative experiences and labeling on the youth can be eliminated, and they will be more ready to resume social engagement and participation. This is related to the enhancement of *psychological capital* (10), and its concepts are illustrated in the subsequent section.

### 2.2. Concepts of psychological capital

Psychological capital (PsyCap), drawn from positive psychology and “positive organizational behavior” [(10), p. 46], is defined as “an individual’s positive psychological state of development” featured by: (1) “having confidence (self-efficacy) to adopt and put in the necessary effort to succeed at challenging tasks”; (2) “making a positive attribution (optimism) about succeeding now and in the future”; (3) “persevering toward goals and, when necessary, redirecting paths to goals (hope) to succeed”; and (4) “when beset by problems and adversity, sustaining and bouncing back and even beyond (resiliency) to attain success” (24). Simply put, psychological capital captures the concepts of *self-efficacy*, *optimism*, *hope*, and *resilience* that constitute one’s success. Self-efficacy, originated from Bandura (25), means “individual’s conviction… about his or her abilities to mobilize the motivation, cognitive resources, and courses of action needed to successfully execute a specific task within a given context” [(26), p. 66]. Optimism, refers to “generalized expectancy for favorable outcomes,” attributing events as positive and positively anticipating life events [(27–29), p. 174]. Hope refers to a positive motivational state based on an interactively derived sense of successful (a) agency (goal-oriented energy) and (b) pathways (planning to meet goals)” [(30), p. 287], whereas agency refers to the belief of being able to create influence (25) and pathways refer to one’s ability to establish ways to attain a certain goal (31). Resilience refers to one’s “capacity to ‘bounce back’ from adversity,” upholding strong beliefs in the meaningfulness of life and responding to changes [(10, 32), p. 47]. Referring to one’s psychological capacities defining “who you are,” psychological capital distinguishes itself from other concepts of capital such as human capital (i.e., “what you know,” defined by one’s knowledge and skills), and social capital (i.e., “who you know,” defined by the capital that can be acquired from social networks) [(10), p. 46].

Originally, psychological capital constituted positive psychological capacities related to performance enhancement, such as “higher productivity,” “better customer service,” “more employee retention,” and “job satisfaction” [(33), p. 127; (10), pp. 46–47]. Applying this concept to general life situations, it can be regarded as “a viable set of resources and mechanisms that can promote the well-being” of individuals in the face of adversities in life [(34), p. 180]. Being able to appraise events positively and maintain perseverance when facing challenges promotes adaptive problem-solving, enhances creativity, helps alleviate stress and negative emotions (e.g., depression and anxiety), enhances positive emotions, enhances psychological well-being, and life satisfaction in different domains (work, relationships, and health) (17, 33, 35–37). In contrast, a low level of psychological capital leads to high susceptibility to emotional distress and a low sense of meaning in life (38, 39).

### 2.3. Enhancing the psychological capital of hikikomori: using different therapeutic approaches

Based on the needs of hikikomori informed by existing literature and the researcher’s own direct practice of hikikomori, researchers implement cognitive-behavioral therapy (CBT), narrative therapy (NT), and play therapy for hikikomori, aiming to enhance their sense of self and develop their capabilities to deal with issues in daily life (see Table 1). The following sections illustrate the application of these three therapies for hikikomori and their relevance to psychological capital.

**Table 1:** Therapeutic intervention received by the participants.

Therapy	Therapeutic goal	Aims/contents	
1. Cognitive-behavioral therapy (Individual and group)	To deal with the irrational beliefs behind hikikomori’s hidden behavior	Session 1	To identify the irrational beliefs (e.g., “Escape is the best way of coping”, “No one cares about my feelings”), with daily life examples sharing Homework assignment (automatic thoughts)
		Session 2	
		Session 3	
		Session 4	To find out alternative ways of coping Homework assignment (coping skills)
		Session 5	
		Session 6	To set goals and develop action plans for change
		Sessions 7-12 (Follow-up sessions)	Flash mind reminding (through Whatsapp): To further consolidate the learning from the intervention Assignments for keeping track the participants’ changes
2. Narrative therapy (Individual and group)	To help hikikomori enhance their sense of self	*The number of sessions held: depending on individual’s situations	Externalization: To deconstruct the life stories of hikikomori
			To find out the unique outcomes from the life experiences To form alternative stories and find their self-preferred identities:
			Outsider witness group (formed by hikikomori’s significant others: To reinforce the self-preferred identities and the alternative stories)
			Flash card reminding (formed by the hikikomori and their significant others: To reinforce the self-preferred identities and the alternative stories)
			Therapeutic documents: To record own thoughts and experiences throughout the intervention, and enhance insights and reflection after reviewing these documents
			Ceremony and certificate: To celebrate the growth and changes of hikikomori
3. Play therapy (Group)	To help hikikomori learn their social norms, rules, and regulations	e.g., Circle time (held weekly): Issues observed during hikikomori’s online gaming (e.g., frequent loss in activities due to individual behavior of hikikomori, such as not being able to comply the rules and regulations in the game) Issues brought into discussion among hikikomori participants Practitioner using these issues as opportunities to help hikikomori understand the rationale behind complying with the rules and learn how to comply with them	
	To help hikikomori acquire various capabilities (e.g., social skills, problem-solving skills, analytical skills, leadership skills) and find their preferred identities	Online game activities (regular, no specific numbers of sessions), e.g.: MVP: To beat the boss monsters, and increase reputation in the game Hikikomori forming groups in the game Practitioner participating in the activity, facilitating the hikikomori’s gaming, and observing the process Debriefing Capture Territories: To increase reputation and resources for own guild Hikikomori forming guilds in the online gaming platforms War between the guilds Practitioner participating in the activity, facilitating the hikikomori’s gaming, and observing the process Debriefing	

#### 2.3.1. Cognitive-behavioral therapy

Cognitive behavioral therapy (CBT), a therapeutic approach combining behavioral and cognitive therapies (40), has an underlying philosophy of how people think, perceive, interpret, and make judgments (i.e., B, “belief”) in situations (i.e., A, “activating event”) that impact their emotional experiences (i.e., C, “consequence”) (13). It focuses on examining one’s automatic thoughts and evaluative judgments aroused unconsciously when faced with situations, which affects one’s emotional, physiological, and behavioral reactions (13). Distorted thoughts result in individuals’ over-attribution of events to the negative side and faulty interpretations of reality (41). On this basis, this therapy aims to help people identify and make changes in their disruptive thinking patterns to minimize the occurrence of negative emotions and behaviors, so as to enhance their problem-solving abilities (13). To achieve this end, therapeutic techniques include both cognitive (e.g., functional analysis, cognitive restructuring, Socratic questioning) and behavioral elements (e.g., thought recording, cognitive rehearsal, activity monitoring, and scheduling) (12, 13, 42). Existing research has found that cognitive-behavioral therapy is effective in enhancing psychological capital, such as in the contexts of divorce and domestic violence (43, 44).

#### 2.3.2. Narrative therapy

Narrative therapy, developed by Michael White and David Epston (45), stresses the importance of narratives in shaping one’s accounts and the expression of problems (46). Since a person’s meaning creation in terms of events and situations are largely influenced by sociocultural norms and factors (47), the therapy deconstructs the meaning of the client’s narratives and “help people identify dominant problem-saturated narratives, discover exceptions to these narratives, and generate alternative preferred stories to ‘re-author’ their lives” [(48), p. 190; (49)], to enhance their sense of self and psychological well-being [e.g., (50)]. The techniques employed to attain such a therapeutic goal include externalization, finding “unique outcomes,” “re-authoring,” “re-membering,” “outsider witnessing,” and using therapeutic documents [(48, 51), p. 192; (52, 53), pp. 61, 62, 129, 164–165, 219; (14)]. Existing research has found that narrative therapy helps enhance self-efficacy, hope, optimism, and resilience, suggesting that it is related to the enhancement of psychological capital [e.g., (54–57)].

#### 2.3.3. Play therapy

Play therapy, as defined by the Association for Play Therapy (58), refers to “the systematic use of a theoretical model to establish an interpersonal process,” wherein therapists utilize “the therapeutic powers of play to help clients prevent or resolve psychological difficulties” [(59), p. 178]. This therapy is commonly used in children, as their abstract thinking has not yet developed, which affects their expression of complex thoughts and emotions (60). In this sense, the use of play in play therapy is regarded as “the vehicle for communication between the child and the therapist, on the assumption that children will use play materials to directly or symbolically act out feelings, thoughts, and experiences that they are not able to meaningfully express through words” [(61), p. 376]. Regarding choice of toys, criteria for toy selection include whether the toys can (1) engage the clients’ interests, (2) help clients explore real-life experiences, and (3) help clients engage in self-exploration and self-understanding, develop self-control, and express their needs and feelings (62). For example, aggressive puppets (e.g., lions and tigers) and toy soldiers help release aggression, while sand and clay facilitate the creative expression of feelings (63). The application of play therapy can exhaust a wide range of approaches [e.g., (64, 65)] such as psychoanalytic play therapy, structural play therapy, and child-centered/non-directive play therapy (66). Non-directive play therapy is regarded as the foundation of play therapy (67). In this kind of play therapy that aims to help the child deal with their emotional issues and enhance skill development (68, 69), the therapist upholds the following principles: (1) establishing “good rapport” with the child; (2) accepting “the child exactly as” they are; (3) allowing the child to “express” their “feelings completely”; (4) acknowledging “the feelings the child is expressing” and reflecting the feelings to them so that they can acquire insights into their behavior; (5) acknowledging the child’s capabilities to cope with problems; (6) allowing the child to direct the therapeutic process; (7) accepting that therapy is a “gradual process,” thus not hurrying the therapeutic process; and (8) establishing limitations only for the child to learn about his responsibility in the therapeutic process [(70), pp. 73–74], such as not displaying inappropriate behavior (e.g., violence). Regardless of the differences in its implementation, the underlying premise “that connects all play therapies is the focus on both the process of play” and the relationship between the therapist and the client [(64), p. S82]. Despite the wide application of this therapy on children, it can be used to treat different age groups, including adolescents and adults (71, 72). Its therapeutic goal is “to achieve optimal growth and development” (73). “Providing the means for insights, learning, problem-solving, coping, and mastery” [(61), p. 377], play therapy is relevant to the enhancement of psychological capital. Fall’s (74) study found that play therapy was related to enhanced self-efficacy, while the study by Author found that online play therapy, with the effect of empowerment, was significantly related to psychological capital when applied to hikikomori to help them develop various capabilities, such as analytical and problem-solving skills through online gaming.

### 2.4. Insights from the above literature

The above literature illustrates the importance of enhancing the psychological capital of hikikomori and the potential of cognitive-behavioral, narrative, and play therapies in enhancing psychological capital. In the context of hikikomori, despite that existing literature shows the application of cognitive-behavioral therapy, narrative therapy, and play therapy on hikikomori [e.g., (15, 75, 76, 77)], the linkage between these therapies and psychological capital is scarcely researched. Regarding the therapeutic outcomes of cognitive-behavioral therapy. Much of the literature focuses on how cognitive-behavioral therapy deals with the cognitive aspects (e.g., “psychoeducation,” “cognitive restructuring,” “emphasizing the relationship between dysfunctional belief systems and behavioural avoidance”), behavioral aspects (e.g., “behavioral activation routines and planning of outdoor social activities”), and social relationships of the hikikomori [(76), p. 74; (77), p. 455] rather than psychological capital. For the therapeutic outcomes of narrative therapy and play therapy for hikikomori, they are even underresearched; there is only one study investigating the relationship of play therapy with empowerment and psychological capital in the context of hikikomori (15). The study of the linkage between therapies and psychological capital is important for hikikomori, because it is relevant to the goal of intervention for these youth in terms of quality of life, empowerment, and the re-establishment of social roles and lives (3, 78). Hence, this study sought to fill this research gap by assessing the therapeutic outcomes of these three forms of therapy in terms of psychological capital. Besides, cognitive-behavioral, narrative, and play therapies are three different types of therapies with contrasting approaches. For instance, cognitive-behavioral therapy is a directive therapeutic approach in which the therapist adopts “a more instructive stance and to be more active in the process” to achieve the completion of certain tasks [(79), p. 58], whilst narrative therapy is a non-directive therapy in which the therapist adopts a “non-directive therapeutic positioning” and collaborates with the client to foster their changes [(80), p. 501]. Play therapy is also a non-directive approach wherein the therapist ventures into the gaming platform that is familiar to the youth and encourages them “to identify and bring to the session what they wish” (81, p. 19). The nature of therapeutic interventions, such as different levels of directiveness, might influence therapeutic outcomes [e.g., (82, 83)]. Against this backdrop, a simultaneous comparison of their therapeutic outcomes is also worthy of investigation, to determine which therapy is mostly related to hikikomori’s psychological capital. It is expected that corresponding implications regarding service development for hikikomori can be generated.

### 2.5. Present study

Based on the rationale of this study, it aims to achieve the following research objectives:

1. Investigate whether cognitive-behavioral therapy, narrative therapy, and play therapy were related to psychological capital in the context of hikikomori;
2. Investigate whether cognitive-behavioral therapy, narrative therapy, and play therapy differed in therapeutic outcomes in psychological capital in the context of hikikomori.

**Figure 1:**
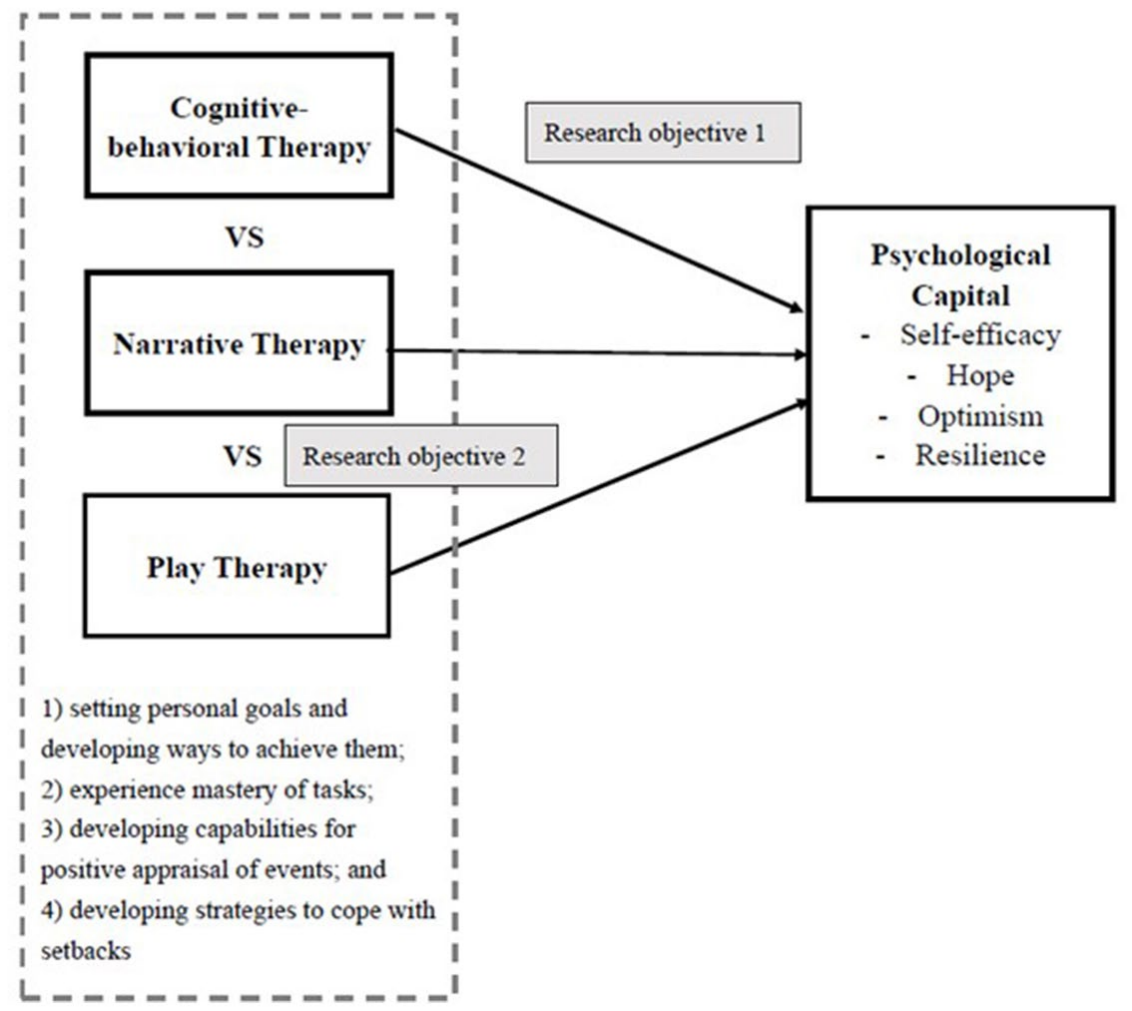
Research framework of this study.

In line with the research objectives, the study hypotheses were as follows:

1. Cognitive-behavioral therapy, narrative therapy, and play therapy were significantly related to the enhancement of psychological capital in the context of hikikomori;
2. Cognitive-behavioral therapy, narrative therapy, and play therapy significantly differed in therapeutic outcomes of psychological capital in the context of hikikomori (see Figure 1).

## 3. Methodology

### 3.1. Study design

To attempt the aforementioned research questions and hypotheses, this study adopted a quantitative research design, using a sample of hikikomori from the Chinese context. The researcher, who had received professional training in social work and counseling, provided therapeutic intervention for them based upon the assessment of their needs and situations. Cognitive-behavioral therapy was provided to help the hikikomori identify irrational beliefs (e.g., “Escaping from the problems is the best way to deal with problems,” “No one cares about by feelings”) underlying their social withdrawal behavior and learn about the alternative ways to solve problems (12, 13); narrative therapy was provided for the hikikomori who developed a negative sense of self and identity due to the severe negative labeling by the mainstream society (14); play therapy was provided for the hikikomori as a way to engage their interests and help them achieve all-round personal development (e.g., social skills, problem-solving skills) (15). In accordance with the participants’ unique needs and situations, some participants received one kind of therapy only, while others received more than one type of therapy (e.g., narrative therapy + play therapy, narrative therapy + cognitive-behavioral therapy, all the three kinds of therapies) (see Tables 1, 2). The effectiveness of these three kinds of therapeutic intervention on psychological capital was investigated through quantitative methods.

**Table 2:** Demographic background of the participants (*N* = 502).

Variables	*N* (%)
Gender	
Male	384 (76.5%)
Female	118 (23.5%)
Age	
12–14	69 (13.8%)
15–17	216 (43.0%)
18–21	217 (43.2%)
Education background	
Secondary 1 to 3	74 (14.7%)
Secondary 4 to 6	277 (55.2%)
College/university	151 (30.1%)
Case recruitment	
Referrals from other agencies and organizations	43 (8.6%)
Parents who enquired for support	70 (13.9%)
Hikikomori who sought help by themselves	122 (24.3%)
Engagement by social workers	256 (51.0%)
Others	11 (2.2%)
Level of social withdrawal	
Level 1	87 (17.3%)
Level 2	281 (56.0%)
Level 3	90 (17.9%)
Level 4	18 (3.6%)
Level 5	26 (5.2%)
Therapy/therapies received	
Narrative therapy only	22 (4.4%)
Cognitive-behavioral therapy only	59 (11.8%)
Play therapy only	47 (9.4%)
Narrative therapy + cognitive-behavioral therapy	3 (0.6%)
Narrative therapy + play therapy	103 (20.5%)
Cognitive-behavioral therapy + play therapy	101 (20.1%)
All therapies	167 (33.3%)

### 3.2. Participants

All of the participants were eligible for the study based on the following inclusion and exclusion criteria: (1) being Hong Kong residents; (2) having socially withdrawn for at least 6 months; (3) being within the age range of 12–30 years old, as it fitted into the age criterion of youth [e.g., (84)]; and (4) not having been diagnosed as having psychiatric illnesses. This was based on the definition of hikikomori that it is “a condition where a youth withdraws into the home and does not participate in society for a period of over 6 months, of which a mental illness is not likely to be the primary cause” [(1), p. 25]. Reviewing the existing literature, there existed two perspectives of hikikomori: Psychiatric perspective and non-psychiatric/sociological perspective (85). While the psychiatric perspective of hikikomori sees it as associating with mental illnesses such as social anxiety and mood disorders [e.g., (86–88)], the non-psychiatric/sociological perspective views hikikomori as a state, behavioral pattern, or lifestyle rather than a mental illness, and it is caused by societal factors (e.g., labeling), school factors, and family factors [e.g., (20, 89, 90)]. Due to the complicated causes of hikikomori, this study adopted the latter perspective in defining hikikomori, in which the intervention focused on dealing with the social withdrawal behavior. Intervention on hikikomori with mental illnesses not only involves the handling of their social withdrawal behavior but also the treatment for their mental illnesses, which will increase the variances of the intervention and the outcomes with regard to the dealing with social withdrawal behavior.

The participants of the study, who were cases of a local service center founded by the researcher, were mainly recruited through: (1) referrals from other agencies and organizations; (2) parents who inquired for support; (3) hikikomori who sought help by themselves; and (4) engagement by social workers. In this regard, participants in this study were recruited *via* convenience sampling, based on the accessibility and availability of the participants (91).

### 3.3. Data collection procedure

After the purpose and procedures of the study had been explained to the participants, the participants provided informed consent. Parental consent was obtained from participants aged below 18 years. After that, each participant was invited to fill in a questionnaire regarding their self-perceived service outcomes in a face-to-face manner. Information about the services which participants received was reported by researcher based on the attendance records for intervention kept by the researcher.

### 3.4. Measurements and scales

#### 3.4.1. Demographic variables

They included age, gender, education level, and level of social withdrawal. Regarding the level of social withdrawal, it indicates the severity of the participants’ social withdrawal state. According to Oiwa (92), there are five levels of social withdrawal, identified based on the amount of social relationships and social support that the hidden youth have: (1) “In the past 3 months, I have not gone outside”; (2) “In the past 3 months, I have not engaged in face-to-face connections”; (3) “In the past 3 months, I have only stayed at home”; (4) “In the past 3 months, I have only stayed in my room”; and (5) “In the past 3 months, I have not talked to anybody.” Whereas level 1 represents the lowest level of social withdrawal, level 5 indicates the most severe level of social withdrawal and the lowest level of social engagement.

#### 3.4.2. Treatment variables

These variables included variables for cognitive-behavioral therapy, narrative therapy, and play therapy, which indicated participants’ receipt for these therapies. Regarding the *cognitive-behavioral therapy* variables, there were 7 self-constructed items. Apart from individual counseling and group counseling, services aiming at helping participants, identify their irrational beliefs, find alternative ways, and make plans were provided. Additionally, reminder messages to reinforce therapeutic outcomes were sent through online communication platforms (e.g., WhatsApp). Regarding the *narrative therapy* variables, there were 13 self-constructed items. These included individual counseling, group counseling, outsider witness, therapeutic documents, autography, ceremony, certificate, thankful group, and flash card reminders (implemented through online communication platforms such as WhatsApp to reinforce the therapeutic outcomes). Regarding the *play therapy* variables, there were 18 self-constructed items. Eleven of them were online games (e.g., “MVP (RPG game),” “Capture territories (RPG game),” “Monopoly in FB (board game),” “Sims online (role play)”), and seven were offline games (e.g., board games, emotional Bingo, activity group). All of the aforementioned items were rated on a 2-point scale (0 = “No,” 1 = “Yes”), which indicated whether the participants had participated in the therapeutic activities.

#### 3.4.3. Psychological Capital Questionnaire (PCQ-24)

This 24-item scale was used to assess the participants’ psychological capital (93) (*α* = 0.95). The items were categorized into four subscales, namely, *self-efficacy* (6 items; e.g., “I feel confident analyzing a long-term problem to find a solution”), *hope* (6 items; e.g., “There are lots of ways around any problem”), *resilience* (6 items; e.g., “When I have a setback at work, I have trouble recovering from it and moving on.”), and *optimism* (6 items; e.g., “I am optimistic about what will happen to me in the future as it pertains to work”). The items were adapted to suit the context of hikikomori. They were rated on a 6-point Likert scale (1 = “strongly disagree;” 6 = “strongly agree”). Negatively phrased items were reverse-scored to ensure their internal consistency. Construct validity of the scale was confirmed in previous studies, on various age groups (e.g., youth, young adults) [e.g., (94, 95)].

### 3.5. Statistical analyses

SPSS 26 was used to perform the statistical analyses. To investigate whether cognitive-behavioral therapy, narrative therapy, play therapy, and psychological capital were related to the psychological capital of the participants, a hierarchical linear regression analysis (Enter method) was conducted to investigate the effect of therapeutic intervention on psychological capital, controlling the effects of participants’ demographic background. By entering the demographic variables (age, gender, education level, and level of social withdrawal) as a block in Level 1, and entering “therapeutic intervention” in Level 2, it helped understand the significance of therapeutic intervention in affecting psychological capital, beyond the demographic variables. Also, Spearman’s rank correlation analysis was performed to investigate the relationship between the three therapies with psychological capital. Subsequently, One-way ANOVA was performed to compare the means of the three therapies in psychological capital to investigate whether these therapies had significant differences in their relationships with psychological capital. To determine how these therapies differed in their relationship with psychological capital, instead of performing multiple independent samples t-tests, the Bonferroni correction was used as the post-hoc analyses for the multiple comparisons testing by comparing the mean scores for psychological capital (overall and subscales) among the three therapies, so as to reduce error rates. This helps shed light on which therapy performed best in the outcome of psychological capital and how they worked.

## 4. Results

### 4.1. Demographics of the participants

A total of 502 participants with hikikomori participated in this study. 502 valid questionnaires were received with no missing data. Table 2 presents the demographic data of the participants. Over 70% of them were male (76.5%, *N* = 384), while the rest were female (23.5%, *N* = 118). Participants were aged between 12 and 21 years, and most of them (86.2%, *N* = 433) were aged 15 years or above. In addition, most of them (85.3%, *N* = 428) had an education level of Secondary 4 to 5 or above. Regarding their level of social withdrawal, around half of them were in level 2 of social withdrawal (56%, *N* = 281). For the recruitment of cases, they were mainly engaged by social workers (51%, *N* = 256), while others were hikikomori seeking help themselves (24.3%, *N* = 122), parents who inquired for support (13.9%, *N* = 70), or referrals from other agencies and organizations (8.6%, *N* = 43) (see Table 2).

### 4.2. Whether the three therapies were related to psychological capital

To begin with, as shown in the hierarchical linear regression analysis, participants’ demographic variables (age, gender, education level, and level of social withdrawal) were entered as Level 1, whereas therapeutic intervention (cognitive-behavioral therapy, narrative therapy, and play therapy which were received by the participants based on their needs and situations) was entered as Level 2. Results showed that demographic background displayed no significant effects on psychological capital, except level of social withdrawal (*p* < 0.05). In Level 1, gender (*β* = −0.10, *p* < 0.05) and level of social withdrawal (*β* = −0.22, *p* = 0.0000) were positively related to psychological capital. However, when therapeutic intervention was entered into Level 2, the effect of gender on psychological capital became insignificant. For level of social withdrawal, although it was still significantly related to psychological capital in Level 2 (*β* = −0.09, *p* < 0.05), its effect on psychological capital was greatly diminished. In Level 2, therapeutic intervention displayed a significant positive effect on psychological capital (*β* = 0.62, *p* = 0.0000); also, the effect of therapeutic intervention on psychological capital was much higher than that of gender, as shown from the regression coefficients. The inclusion of therapeutic intervention in Level 2 contributed to a change in variance in psychological capital from 7 to 42% (Δ*R*^2^ = 0.35). This reflects that the use of therapies (cognitive-behavioral therapy, narrative therapy, play therapy) was significantly related to the enhancement of psychological capital (see Table 3).

**Table 3:** Hierarchical linear regression analysis predicting hikikomori’s psychological capital.

	Model 1		Model 2	
	*ß*	SE	*ß*	SE
Demographic variables				
Age	0.04	0.04	−0.06	0.03
Gender	−0.10*	0.10	−0.04	0.08
Education level	−0.13	0.11	0.05	0.09
Level of social withdrawal	−0.22**	0.05	−0.09*	0.04
Therapeutic intervention			0.62**	0.02
*R*	0.27		0.65	
Adjusted *R*^2^	0.07		0.42	
*F*	10.12		73.43	
Sig.	0.0000		0.0000	

*p < 0.05, **p < 0.001.

For the results of the correlation analysis (*N* = 502), based on Dancey and Reidy’s (96) naming of the magnitude of the correlation coefficients, it showed that cognitive-behavioral therapy (*r* = 0.768) was significantly strongly positively correlated with psychological capital (total score) with a high level of statistical significance (*p* = 0.0000), whereas play therapy (*r* = 0.633) and narrative therapy (*r* = 0.467) was significantly moderately positively correlated with psychological capital (total score) with a high level of statistical significance (*p* = 0.0000). Additionally, the three therapies were significantly positively correlated to the four subscales of psychological capital (self-efficacy, hope, resilience, optimism), in various magnitudes (see Table 3). This indicates that these three forms of therapy are related to the enhancement of psychological capital (Table 4).

**Table 4:** Inter-variable correlations of all measurement scales (*N* = 502).

	Psychological capital (overall)	Psychological capital (self-efficacy)	Psychological capital (hope)	Psychological capital (resilience)	Psychological capital (optimism)
Cognitive-behavioral therapy	0.768**	0.806**	0.654**	0.802**	0.467**
Narrative therapy	0.467**	0.338**	0.566**	0.290**	0.625**
Play therapy	0.633**	0.440**	0.686**	0.368**	0.651**

** *p* < 0.001.

### 4.3. Whether the three therapies had significant differences in psychological capital

In One-way ANOVA, participants receiving cognitive-behavioral therapy only, narrative therapy only, and play therapy only were selected for comparison (*N* = 128). The results showed that there were significant differences among the three therapies in the total score of psychological capital, *F*(2, 125) = 17.92, *p* = 0.0000, as well as its subscales of self-efficacy, *F*(2, 125) = 70.83, *p* = 0.0000, resilience, *F*(2, 125) = 38.39, *p* = 0.0000, and optimism, *F*(2, 125) = 11.62, *p* = 0.0000. The mean psychological capital for the cognitive-behavioral therapy group was the highest in the total score of psychological capital and so as its subscales of self-efficacy and resilience, while narrative therapy only scored the highest in the subscale of optimism. There were no significant differences in the three therapies in the subscale of hope (see Table 5). This reflects that the three therapies may work differently in the enhancement of psychological capital.

**Table 5:** Results of the One-way ANOVA in psychological capital for the three groups cognitive-behavioral therapy only, narrative therapy only, and play therapy only (*N* = 128).

	Cognitive-behavioral therapy only (*N* = 59)		Narrative therapy only (*N* = 22)		Play therapy only (*N* = 47)			
	*M*	SD	*M*	SD	*M*	SD	*F*	*p*
Psychological capital (overall score)	2.75	0.77	2.22	0.52	2.05	0.43	17.92	0.0000
Psychological capital (self-efficacy)	3.19	0.88	1.64	0.55	1.74	0.48	70.83	0.0000
Psychological capital (hope)	2.42	0.75	2.55	0.64	2.22	0.54	2.28	0.107
Psychological capital (resilience)	3.38	1.48	1.66	0.56	1.73	0.48	38.39	0.0000
Psychological capital (optimism)	2.03	0.89	3.02	0.94	2.52	0.77	11.62	0.0000

### 4.4. How the three therapies were related to psychological capital (overall and subscales): a comparison

#### 4.4.1. “Cognitive-behavioral therapy only” vs. “narrative therapy only” vs. “play therapy only”

Post-hoc comparisons using the Bonferroni correction were performed to compare the outcomes in psychological capital (overall scale and subscale) between “cognitive-behavioral therapy only,” “narrative therapy only,” and “play therapy only” groups. Results showed that the “cognitive-behavioral therapy only” group scored significantly higher than the “narrative therapy only” group (*p* < 0.05) and the “play therapy only” group (*p* = 0.0000) in the overall score for psychological capital, and its subscales “self-efficacy” and “resilience” (*p* < 0.05). The “narrative therapy only” and “play therapy only” groups showed no significant differences in the overall score for psychological capital and its four subscales. In addition, in the “optimism,” subscale, the “narrative therapy only” (*M* = 3.02, SD = 0.94) group showed significantly higher score than the “cognitive-behavioral therapy only” group (*M* = 2.03, SD = 0.89) (*p* = 0.0000), and the “play therapy only” (*M* = 2.52, SD = 0.77) group showed significantly higher score than the “cognitive-behavioral therapy only” group (*M* = 2.03, SD = 0.89) (*p* = 0.0000) (see Table 6). This reflects that cognitive-behavioral therapy worked best in enhancing the participants’ psychological capital in terms of self-efficacy and resilience, while narrative therapy worked best in hope and optimism than cognitive-behavioral therapy.

**Table 6:** Bonferroni post-hoc comparisons between psychological capital and different kinds of therapies (cognitive-behavioral therapy only, narrative therapy only, and play therapy only) (*N* = 128).

	Cognitive-behavioral therapy only (*N* = 59)		Narrative therapy only (*N* = 22)		Play therapy only (*N* = 47)		Post-hoc comparisons	
	*M*	SD	*M*	SD	*M*	SD	Mean difference	*p*
Psychological capital (overall score)	2.75	0.77	2.22	0.52	–	–	0.54	0.002
	2.75	0.77	–	–	2.05	0.43	0.70	0.0000
	–	–	2.22	0.52	2.05	0.43	0.17	0.901
Psychological capital (self-efficacy)	3.19	0.88	1.64	0.55	–	–	1.55	0.0000
	3.19	0.88	–	–	1.74	0.48	1.45	0.0000
	–	–	1.64	0.55	1.74	0.48	−0.10	1.000
Psychological capital (hope)	2.42	0.75	2.55	0.64	–	–	−0.13	1.000
	2.42	0.75	–	–	2.22	0.54	0.20	0.351
	–	–	2.55	0.64	2.22	0.54	0.34	0.154
Psychological capital (resilience)	3.38	1.48	1.66	0.56	–	–	1.72	0.0000
	3.38	1.48	–	–	1.73	0.48	1.65	0.0000
	–	–	1.66	0.56	1.73	0.48	−0.07	1.000
Psychological capital (optimism)	2.03	0.89	3.02	0.94	–	–	−0.99	0.0000
	2.03	0.89	–	–	2.52	0.77	−0.48	0.014
	–	–	3.02	0.94	2.52	0.77	0.51	0.072

#### 4.4.2. Receiving multiple therapies of cognitive-behavioral therapy, narrative therapy, and play therapy: a comparison to single therapies

Since some participants received more than one therapy, One-way ANOVA, with post-hoc comparisons using the Bonferroni correction, was performed to compare the outcomes of psychological capital (overall scale and subscale) between multiple therapies group (“narrative therapy + play therapy,” “narrative therapy + cognitive-behavioral therapy,” “cognitive-behavioral therapy + play therapy”) and single therapies group (“narrative therapy only,” “play therapy only,” and “cognitive-behavioral therapy”), to see whether combining the features of different therapies would bring about better outcomes in psychological capital. The results showed that the “cognitive-behavioral therapy + play therapy” performed better than the “cognitive-behavioral therapy only” group in psychological capital (overall score) and its subscales of self-efficacy and resilience (*p* = 0.0000). Besides, the “narrative therapy + play therapy” group performed better than the “narrative therapy only” group in the subscales of “hope” (*p* = 0.0000) and “optimism” (*p* < 0.05) of psychological capital (see Table 7). This suggests that integrating play therapy in the intervention enhances the outcomes of narrative therapy and cognitive-behavioral therapy in psychological capital.

**Table 7:** Results of the One-way ANOVA in psychological capital and different kinds of therapeutic interventions with Bonferoni post-hoc comparisons (*N* = 335).

	1. Narrative therapy only (*N* = 22)		2. Play therapy only (N = 47)		3. Cognitive-behavioral therapy only (*N* = 59)		4. Narrative therapy + play therapy (*N* = 103)		5. Play therapy + cognitive-behavioral therapy (*N* = 101)		6. Narrative therapy + cognitive-behavioral therapy (*N* = 3)		Post-hoc comparisons	
	*M*	SD	*M*	SD	*M*	SD	*M*	SD	*M*	SD	*M*	SD	Mean difference	*p*
Psychological capital (overall score)	2.22	0.52	2.05	0.43	2.75	0.77	3.13	0.89	3.75	0.65	2.63	1.91	5–3: 0.99	0.0000
Psychological capital (self-efficacy)	1.64	0.55	1.74	0.48	3.19	0.88	2.78	1.14	4.27	0.90	2.61	1.21	5–3: 1.08	0.0000
Psychological capital (hope)	2.55	0.64	2.22	0.54	2.42	0.75	3.32	0.97	3.42	0.64	1.39	0.42	4–1: 0.77	0.0000
Psychological capital (resilience)	1.66	0.56	1.73	0.48	3.38	1.48	2.77	1.12	4.26	0.89	5.50	6.06	5–3: 0.88	0.0000
Psychological capital (optimism)	3.02	0.94	2.52	0.77	2.03	0.89	3.65	0.89	3.05	0.74	1.00	0.00	4–1: 0.63	0.019

5–3: Mean differences between “play therapy + cognitive-behavioral therapy” and “cognitive-behavioral therapy only.”

4–1: Mean differences between “narrative therapy + play therapy” and “narrative therapy only.”

## 5. Discussion

This study investigated the relationship between therapies (cognitive-behavioral, narrative, and play therapies) and psychological capital in the context of hikikomori. Also, due to the differences in the approaches between these therapies (e.g., directive VS. non-directive), this study simultaneously compared their outcomes of psychological capital. Correlation results showed that cognitive-behavioral therapy, narrative therapy, and play therapy were positively related to psychological capital, reflecting that these three therapies are related to the enhancement of psychological capital in the context of hikikomori. Meanwhile, results of the One-way ANOVA showed that the three therapies had significant differences in psychological capital (except the subscale of hope). Post-hoc analyses showed that cognitive-behavioral therapy scored significantly higher in psychological capital (overall scale as well as subscales of “self-efficacy” and “resilience”) than the other two therapies, whilst in the subscale of “optimism,” narrative therapy and play therapy scored significantly higher than cognitive-behavioral therapy, respectively. Besides, when comparing the use of multiple therapies with single therapies, results of the One-way ANOVA with post-hoc analyses showed that the “cognitive-behavioral therapy + play therapy” group exhibited better outcomes in psychological capital (overall scale, as well as subscales of “self-efficacy” and “resilience”) than the “cognitive-behavioral therapy only” group, and the “narrative therapy + play therapy” group displayed better outcomes in “hope” and “optimism” than the “narrative therapy only” group. All these results indicate that cognitive-behavioral therapy, narrative therapy, and play therapy had varied strengths, in relation to the context of hikikomori.

Cognitive-behavioral therapy is featured by a directive approach with a specific focus on identification of the clients’ needs, “conscious processes,” “action,” and “solution” [(79), pp. 56–57]. It seeks to “alleviate clients’ distress and enhance their coping and problem-solving abilities with an elaborate, well-planned approach,” so as to “increase their effectiveness in handling their daily lives” [(97), p. 49]. This therapy also entails the concept of psychological capital, which constitutes the “positive resources,” including “the positive cognition, emotions, and behaviors” that help the participants to deal with current emotional problems and enhance their capabilities to cope with future stress and adversities” [(98), p. 4]. Given the nature of being a problem-focused and goal-oriented therapy which specifically deals with participants’ emotions and maladaptive thoughts and foster their behavioral changes, cognitive-behavioral therapy is particularly applicable for hikikomori who withdraw due to maladaptive thoughts and coping patterns (e.g., “Escaping from the problems is the best way to deal with problems” and “No one cares about by feelings.”), and enhancing their adaptation to mainstream society.

For narrative therapy, it is “a strengths-based approach to psychotherapy” which helps “clients see themselves as empowered and capable of living the way they want,” and helps them develop an attitude of “This too will pass” when facing difficulties [(99), p. 695]. The application of this therapy to hikikomori is to reduce their internalization of problems and empower them (99), rather than facilitating their adaptation to the standards of mainstream society and subsequent re-engagement in society. Hence, it is particularly applicable for hikikomori who develop a negative sense of self and identity (e.g., loss, failure, rubbish) in the face of severe negative labeling and censuring from mainstream society due to their inability to fit into the education system and labor market (3). In this sense, narrative therapy is more relevant to the enhancement of hope and optimism than the concept of psychological capital.

Besides, it is found that integrating play therapy with cognitive-behavioral therapy and narrative therapy, respectively, could bring about better outcomes of psychological capital than using either cognitive-behavioral therapy or narrative therapy alone in the context of hikikomori. This may be due to the nature and functions of play therapy. Play therapy is a “non-directive, insight-oriented or facilitative” [(97), p. 47] therapeutic approach aiming at enhancing clients’ self-esteem, regulation of emotions, problem-solving skills, and stress relief (63). It not only is therapeutic itself, but also helps enhance the therapeutic process through facilitating relationship building between the worker and the service user (100), and enhancing participants’ motivation for learning (101). Hikikomori have an immense interest in online activities especially online gaming (3, 15). Integrating play therapy into the therapeutic process could likely engage their interests and serves as an additional support for their growth and development.

## 6. Limitations of this study

This study has several limitations. One important limitation concerns with the therapeutic intervention received by the participants. Since the therapeutic intervention was delivered in accordance with the participants’ specific needs and situations which underlay their social withdrawal state, there were participants who received some other intervention (e.g., solution-focused therapy) apart from the three therapies. Hence, these participants (i.e., the “All therapies group”) were not applicable for comparison. Another important limitation is the small sample size. As all the participants of this study were cases of a service center and they were assigned to therapeutic interventions in accordance with the professional assessment of their unique needs and situations, the numbers of participants were not balanced among different types of therapeutic interventions. This resulted in small sample size for each type of therapeutic intervention. Next, there is a limitation concerning with treatment integrity, which is referred to as “the extent to which treatment was implemented as intended” [(102), p. 148]. In this study, a standardized and independent measure was not used. Furthermore, the self-report measure on psychological capital constituted a limitation as it might lead to bias. In addition, this was a cross-sectional study which could not assess the maintenance of outcomes after a certain period or at different points in time. In other words, the level of psychological capital of participants who had completed the therapeutic intervention for several years and those who had completed the therapeutic intervention for a shorter period might vary, which likely confounded the results.

## 7. Implications

Based on the results as discussed above, implications are generated as follows. Regarding research implications, future studies can be conducted to address the aforementioned limitations. To begin with, in response to the issue of sample size and the use of measure in this study, more robust studies, such as randomized controlled trials (RCT), should be performed on a larger sample size to further assess the significant differences in the effectiveness and outcomes of different therapies. Also, other objective measurements such as assessment by peers and professionals can be adopted to decrease the bias (103). Next, to complement the results of this cross-sectional study, a longitudinal study should be conducted to assess whether similar outcomes can be achieved, taking the sustainability of different types of interventions into account. A qualitative study can be included as well, to understand the participants’ opinions of different kinds of therapies. Additionally, other directions for future studies can include: (1) further research on the effect of different types of intervention on hikikomori, apart from the three therapies in this study; and (2) the investigation of other outcome variables (e.g., social engagement and self-concept), in order to further enrich such comparative studies on therapeutic interventions.

In terms of clinical/practice implications, this study helps provide empirical support for the potential of using cognitive-behavioral therapy, narrative therapy, and play therapy (single and multiple therapies) to enhance hikikomori’s psychological capital, using the sample of Hong Kong. In the context of Hong Kong, intervention for hikikomori are largely focused on (1) career skills building and employment support (e.g., provision of career-related learning activities, vocational training, and internship opportunities); and (2) social skills and social network building, so as to increase their self-confidence and facilitate their re-engagement into society [e.g., (104, 105)]. The results of this study help provide valuable insights in terms of how to enrich the intervention for hikikomori through counseling, so as to strengthen their inner capacities which can support their re-engagement into society. To maximize the effectiveness of counseling for hikikomori, an in-depth assessment of the unique needs and situations of hikikomori which underlie their social withdrawal behavior is prerequisite. This enables the tailoring of intervention plans which are responsive to their specific needs and conditions. The results regarding the comparison between single and multiple therapies support the use of eclectic approaches/integrative therapies. Such mode of intervention enables the openness and flexibility in the use of therapies through acknowledging the features and advantages of each type of therapy, and combine the therapies to adequately address the clients’ issues (106). Being able to get the hikikomori involved in the therapeutic process (e.g., through play therapy), with suitable intervention modality (e.g., through cognitive-behavioral therapy and/or narrative therapy), helps enhance the therapeutic outcomes [e.g., (107, 108)].

## 8. Conclusion

To conclude, among the three kinds of therapies, cognitive-behavioral therapy showed the best overall outcomes on psychological capital as well as dimensions of self-efficacy and resilience, whilst narrative therapy showed the best outcomes in hope and optimism of psychological capital. Also, using multiple therapies brings better outcomes than using single therapies. Integrating play therapies into the therapeutic process helps enhance the effectiveness of cognitive-behavioral therapy and narrative therapy on psychological capital. These results reflect that cognitive-behavioral therapy, narrative therapy, and play therapy have different strengths.

## Data availability statement

The original contributions presented in the study are included in the article/supplementary material, further inquiries can be directed to the corresponding author.

## Ethics statement

The studies involving human participants were reviewed and approved by the Ethics Research Committee of City University of Hong Kong. Written informed consent to participate in this study was provided by the participants’ legal guardian/next of kin.

## Author contributions

The author confirms being the sole contributor of this work and has approved it for publication.

## Conflict of interest

The author declares that the research was conducted in the absence of any commercial or financial relationships that could be construed as a potential conflict of interest.

## Publisher’s note

All claims expressed in this article are solely those of the authors and do not necessarily represent those of their affiliated organizations, or those of the publisher, the editors and the reviewers. Any product that may be evaluated in this article, or claim that may be made by its manufacturer, is not guaranteed or endorsed by the publisher.
